# The Effect of Horse Shoeing with Egg Bar Shoes and Shoes with Wedge Pads on the Results of Thermal Imaging of the Equine Distal Limb

**DOI:** 10.3390/ani11061479

**Published:** 2021-05-21

**Authors:** Marta Mieszkowska, Zbigniew Adamiak, Piotr Holak, Joanna Głodek, Ewa Jastrzębska, Katarzyna Wolińska, Marcin Mieszkowski

**Affiliations:** 1 Department of Surgery and Radiology with Clinic, Faculty of Veterinary Medicine, University of Warmia and Mazury, Oczapowskiego 14, 10-719 Olsztyn, Poland; zbigniew.adamiak@wp.pl (Z.A.); piotr-holak@wp.pl (P.H.); j_glodek@wp.pl (J.G.); 2 Department of Horse Breeding and Riding, Faculty of Animal Bioengineering, University of Warmia and Mazury, Prawocheńskiego 2, 10-720 Olsztyn, Poland; e.jastrzebska@uwm.edu.pl (E.J.); wolinska.k@gmail.com (K.W.); 3 Department of Anesthesiology and Intensive Care, Faculty of Medicine, Collegium Medicum, University of Warmia and Mazury in Olsztyn, al. Warszawska 30, 10-082 Olsztyn, Poland; marcinm.mieszkowski@gmail.com

**Keywords:** equine, infrared thermography, digital blood flow

## Abstract

**Simple Summary:**

Thermography is a non-invasive and contact-free imaging method that measures temperature on the surface of the body and determines temperature distribution across the examined surface. The aim of this study was to determine the effect of horse shoeing with egg bar shoes and shoes with wedge pads on hoof temperature measured by thermography. The authors decided to use egg bar shoes and shoes with wedge pads as they are commonly used in the treatment of navicular syndrome in horses. This study was conducted on 16 client-owned warmblood horses. The horses were directed for magnetic resonance imaging (MRI) according to unilateral front limb lameness, specifically associated with the hoof. For thermographic analysis, we took into consideration only one limb that was not lame and that showed no radiological changes. After the application of egg bar shoes, the temperature decreased on the palmar surface of the hoof. After shoeing with wedge pads, the temperature decreased in the dorsal and palmar views. Thermography, despite its great usefulness, is only an indirect method of assessing the blood supply in a given area, so we cannot uncritically conclude about the harmfulness of shoeing.

**Abstract:**

The presented manuscript provides reference for practitioners when measuring normal hoof temperature, as well as controlling the temperature after shoeing with particular shoes. The aim of this study was to determine the effect of horse shoeing with egg bar shoes and shoes with wedge pads on hoof temperature measured by thermography. This was a prospective study conducted on 16 horses. The horses were divided into two groups: horses from group 1 were shod with egg bar shoes, while horses from group 2 were shod with shoes with wedge pads. Thermographic examination was performed below the metacarpophalangeal joint before and one month after shoeing. After shoeing with egg bar shoes, there was a decrease in the median of the minimal temperature in the palmar view. After shoeing with wedge pads, thermography revealed decreased hoof temperature in the dorsal and palmar views. Horse shoes may have a negative impact on the blood circulation and metabolism within the distal part of the limb; however, our study found this only to a minor extent.

## 1. Introduction

It has already been concluded that shoeing forelimbs cause changes in hoof morphology and may increase the risk of lameness over time [1]. The expansion of the hoof might be restricted by the shoes [2], thereby blocking heel movement, caused by displacement of the middle phalanx between the ungular cartilages [3], and reducing mediolateral heel movement [4]. Even gluing the shoe can restrict heel movement, reducing shock absorption and blood pumping in the hoof [5]. However, another report has suggested that heel contraction is a multifactorial problem, and the authors did not confirm that horse shoeing causes heel contraction [6].

Thermography is a non-invasive and contact-free imaging method that measures the temperature on the surface of the body and determines the temperature distribution across the examined surface [7]. Thermal images of the skin surface reflect the metabolic status of the tissues and blood flow in a given area. In healthy horses, the temperature on the surface of the skin is around 5 °C lower than in the internal organs [8]. Some previous studies monitored the skin temperature under various clinical conditions and, simultaneously, underlying circulation [9,10]. Blood flow in high-metabolic-rate organs is intensified, and the surrounding region is warmer. Vein thrombosis, infarction, scars, and oedema decrease the blood supply and lower tissue metabolic rate, which is why the affected area is cooler [8]. Any interruptions in the normal blood flow and temperature distribution on the surface of the body could be indicative of pathological processes. A local rise in body temperature is one of the first symptoms of an inflammatory process [10]. Therefore, temperature measurements can support the early detection of diseases [11]. However, some studies have revealed that thermography may not be helpful in diagnosing chronic lameness due to the fact that superficial temperatures can become normal in chronic states [12].

Thermography has many applications in equine medicine. It is very useful in detecting early signs of tendon injuries, showing the temperature of increased flexor tendons even before clinical signs of injury [10], in combination with pain reaction during palpating tendons [7]. While back pain is one of the most important clinical problems in equine athletes, thermography is considered to be a useful diagnostic tool in searching for the back pathologies that are caused by an improperly fitted saddle [13]. In these cases, thermography can be a reliable method of assessing saddle fit, which can significantly prevent the development of back pathology [14,15,16]. It can also be a valuable tool for the diagnosis of a reflex sympathetic dystrophy-like syndrome [17]. What is more, thermography provides the possibility to monitor the progress of treatment. In a previous study, a comparison of pre- and post-treatment thermographic images showed a temperature change indicative of an autonomic nervous system improvement caused by joint manipulation in multiple spinal joint fixations [18]. Another problem faced by clinicians is damage to the skin under the cast applied to the distal part of the limb. Thermography is a valuable and rapid clinical tool to monitor the development of cast sores, thus enabling faster treatment implementation [19].

Relatively new research has shown the use of thermography to compare the eye temperature and a horse’s response to stress [20,21]. In such studies, temperature measurement was taken at the lacrimal caruncle level, where the result is not affected by hair. These studies were partially correlated with those of Radaelli et al. [22], focusing on measuring the level of stress, heart rate, and cortisol level, and measuring the temperature of various parts of the body—eye, crown, pastern, gluteus, and longissimus dorsi muscles. Interestingly, the results of this study showed a correlation between an increase in the temperature of the left and right eyes and heart rate, indicating a new and interesting application of thermography under training and competition conditions.

In the context of competitions, thermography has also been used to detect illegal procedures, such as neurectomy, or the administration of anesthetics to the lumbar region, which results in a change in the thermographic pattern [23]. Thanks to the use of thermography, it is possible to evaluate the effectiveness of training, and here, a study by Maśko et al. [24] indicates the impact of the use of various auxiliary tools during lunging on the degree of horse involvement in the work. Thus, it has been clearly proven that the use of auxiliary tools is more effective and more activating of the horse compared to moving with a loose head and neck.

There are reports of a negative effect of shoeing on hoof temperature measured by thermography. However, there is no specific scientific research for this. We made an attempt to assess the influence of horse shoeing on the results of the thermographic examination. This may be the beginning of further considerations, checking different types of horseshoes, as well as a comparison with venography. Thermography has the advantage of being non-invasive, which almost completely eliminates any limitations on its use.

The aim of this study was to determine the effect of horse shoeing with egg bar shoes and shoes with wedge pads on hoof temperature measured by thermography. The authors decided to use egg bar shoes and shoes with wedge pads as they are commonly used in the treatment of navicular syndrome in horses [25].

## 2. Materials and Methods

The study was conducted on 16 client-owned warmblood horses. The horses were directed for magnetic resonance imaging (MRI) according to unilateral front limb lameness, specifically associated with the hoof. They were diagnosed with navicular bone structural changes and primary deep digital flexor tendon injury. These were clinical patients, which is why the consent of ethical approval was not necessary. The owners signed their approval for magnetic resonance imaging and thermographic testing. Before each MRI, the horses had to be barefoot, so there was an opportunity to perform thermographic testing of the distal limb. For thermographic analysis, we took into consideration only one limb that was not lame and that showed no radiological changes after performing X-rays focused on the hooves—lateral, oxspring, and skyline views. Due to the results of lame limb MRI, the horses were subjected to orthopedic shoeing—horses with navicular bone disease had egg bar shoes, while horses with ddft tendinopathy had wedge pads. In this study, the authors took into consideration eight horses for each group:

- Group 1—eight horses; the first thermographic examination was performed before shoeing. The animals were then shod with egg bar shoes using the cold technique. Thermography was repeated after a month;
- Group 2—eight horses; the first thermographic examination was performed before shoeing. Subsequently, the horses were shod with wedge pads using the cold technique. After a monthly period of adaptation, thermography was repeated.

Animal handling: Before the examination, the horses were kept in a stable for around 12 h with a relatively stable temperature of 8–12 °C (10 °C on average). Rectal temperature, respiratory rate, and heart rate were measured prior to examination. The horses selected for the study were not sedated because alpha-2 agonists, which are most often used to induce sedation in equine patients, interact with the alpha-1 adrenergic receptors of peripheral vessels and stimulate vascular contraction [26]. The above could distort thermographic measurements.

Thermography: The thermographic examination was performed with the use of an FLIR T250 infrared camera with a 320 × 240 resolution, a spectral range of 7.5–13 µm, and a temperature range of −20 to +350 °C. The images were analyzed in three aspects: dorsal, right palmar, and left palmar. Thermographic images were acquired in the distal limb below the metacarpophalangeal joint (fetlock), focusing on the hoof, the phalanx, and the heel bulbs. All examinations were performed in the stable to reduce the influence of external factors such as wind and sun. The distribution of the temperature inside the hoof capsule was determined.

Statistical analysis: The following parameters were calculated to describe the quantitative variables: median, mean, standard deviation, and minimum and maximum values. Frequency tables were developed for the categorical variables. The distribution of the data was evaluated using the Kolmogorov–Smirnov test. Those values for which *p* < 0.05, analyzed using the Mann–Whitney *U*-test and the Wilcoxon signed-rank test, were considered significant at the level of 0.05.

## 3. Results

### 3.1. Group 1

**Figure 1:**
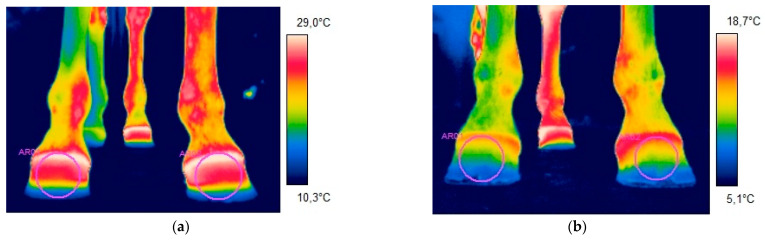
Thermographic image representing the dorsal aspect of the front limbs of a five-year-old Polish halfbred gelding before (**a**) and after (**b**) shoeing with egg bar shoes. The measurements were taken from the left limb, the area of which is marked by a circle. It is clearly visible, based on the color map, that the limb after shoeing was cooler. This was proven with the results of the measurements. The mean temperature decreased from 23.3 °C before shoeing to 5.05 °C after shoeing.

After shoeing with egg bar shoes, the average hoof temperature in the dorsal aspect decreased in the range of 2.8 to 13.8 °C. In the palmar aspect, the difference in temperature did not exceed 1 °C in only 6% of the cases, and in the remaining cases, the temperature decreased in the range of 1.2 to 14.1 °C. Significant differences in temperature (*p* < 0.05) were noted in the median minimum temperature in the palmar aspect, which decreased from 14.088 to 7.8 °C. The results of the thermographic examination before and after shoeing are shown in the Table 1, with a graphic presentation in Figure 1.

### 3.2. Group 2

Interestingly, the minimum temperatures in the various views increased. In all other views, the temperature decreased after shoeing. The detailed results are presented in Table 2.

## 4. Discussion

During the test, it was important to keep stable the environmental conditions prior to the examinations. It has been proven by other authors that ambient temperatures have an impact on limb temperatures measured by thermography [27]. Herein, the ipsilateral temperature differences between limb joints were associated with ambient temperature and were greater when viewing from the lateral and medial sides, but less so from the dorsal aspect.

The horses were also prevented from physical activity 12 h prior to examination, which is crucial for receiving reliable results [28]. During the examinations, the horses were calm, quiet, and stood still, as movement might have influenced the results.

We chose a monthly interval because it has been proven that after eight weeks of shoeing, the center of pressure changes significantly toward the plantar/palmar part of the hoof [29]. We wanted to avoid this phenomenon as much as possible and learn about the impact of shoeing itself, although perhaps waiting longer and changing the position of the hoof as an adaptive response could still have changed the results. Why not immediately after shoeing? We wanted to eliminate the influence of direct manipulations after shoeing, such as introducing shoe nails, trimming hooves, and thinning the horn, so we waited until the horn had grown back a bit and the hooves had adapted; with a relatively small influence of external manipulation factors, we were able to assess the hoof temperature. Particularly importantly, the horses were not examined by MRI immediately after removing the hooves, and most often just about 3–5 weeks after the blacksmith’s visit.

A significant temperature difference is defined as a difference of 0.14–0.39 °C [22]. Turner [7], in turn, assumed that a difference above 1 °C is significant, where modern cameras can detect temperature changes of 0.1 °C. In our study, we did not take into account the differences between the limbs, but we compared the measurement results to those made before shoeing. Although a statistically significant difference concerned the median minimum temperature of the palmar aspect in groups 1 and 2, if one were to look at the individual measurements, this difference exceeded 1 °C, which may indicate a significant influence of shoeing on hoof temperature. Comparing the results of the presented thermographic examinations with venography would greatly increase the value of this publication. Unfortunately, this was not carried out in this case. There is no conclusive information in the scientific literature, apart from some non-scientific studies, regarding the influence of shoeing on hoof blood flow and the results of thermographic examination, which we could discuss. Therefore, we based our conclusions only on our thermographic tests, which are not a direct measurement, but are at least scientifically acceptable as a certain indicator of blood supply in a particular area.

The type of shoes in this study were selected because of their relative popularity in the treatment of equine limb disorders, especially navicular disease and other diseases of the distal part of the limb. The role of wedge pads is to decrease the load on the navicular apparatus by rising the angle to the ground above 70° [30,31]. In turn, the role of egg bar shoes is to shift body weight palmarly to the navicular apparatus and to increase the surface area of the sole [32]. However, it can be expected that shoes may contribute to the compression of laminar vessels, which can impair the hoof mechanism and, in this manner, might result in decreased nutrition of the soft tissues in the distal part of the limb. The influence of proper trimming of the hoof capsule on the position of the coffin bone has been proven [33]. Almost 72% of horses showing lameness of the thoracic limb show radiographic changes of the toe [34]. After shoeing with wedge pads, a change in the position of the distal interphalangeal joint has been observed, which is more flexed, resulting from a steeper position of the limb [35]. The above could increase the pressure exerted by the coffin bone on the circumflex artery in the dorsal aspect of laminar tissue and the reduction in load on the deep digital flexor tendon in this position, which reduces heat generation inside the hoof capsule. This is why, after shoeing with wedge pads, the temperature decreased in the dorsal and palmar views.

After application of egg bar shoes, the temperature decreased on the palmar surface of the hoof. Similar results were reported by Ritmeester et al. [36], who observed that egg bar shoes increase the perfusion in the dorsal portion of laminar tissue.

The minimum temperature decreased due to the presence of horseshoes, and this is important information for us. There was no significant drop in maximum temperature, due to the persistently higher temperature in the coronary band, which suggests that the horseshoe had no effect on blood circulation in this area.

A more significant drop in temperature was noted after shoeing with horseshoes with a wedge pad. In all of the horses in the two groups, the minimum temperature decreased in the palmar aspect. This may indicate impaired circulation in the hoof after the use of shoes. However, thermography, despite its great usefulness, is only an indirect method of assessing the blood supply in a given area, so we cannot uncritically conclude about the harmfulness of shoeing.

## 5. Conclusions

The use of egg bar shoes contributes to a decrease in the median minimum temperature in the palmar aspect of the hoof.

In horses shod with wedge pads, the median minimum temperature decreased in the dorsal and palmar aspects of the hoof.

However, thermography is a diagnostic tool that is helpful in determining indirectly the metabolic status in a specified area, and it is worth remembering that this method is susceptible to the influence of external factors; therefore, examination should be performed under controlled conditions, and the results should be interpreted with a certain degree of caution.

## 6. Limitations

When writing conclusions from the study, the authors did not fail to observe certain limitations. The first limitation of this study is the quite low number of patients. If the horses were to be re-examined after forced movement, perhaps the overall results would have led to additional conclusions. We also studied the temperature, averaged over the entire hoof, in particular views, while less generalized conclusions could be obtained by separating the measurements from the small areas of the hoof. It is important to pay attention to the time after which the next thermographic examination is performed. In this study, we assumed it would be a month. The conditions during the re-examination were similar to those during the first measurements, but do not forget that the weather may change significantly within a few weeks, which will have a significant impact on the measurement results. Additionally, we studied the influence of specific horseshoes on the thermographic tests. The results could be different when using other types of shoes, and therefore the conclusions relate to the shoes used in the study, rather than a generalized conclusion about the effect of shoeing on hoof temperature.

## Figures and Tables

**Table 1:** Thermographic results received from group 1 before and after shoeing with egg bar shoes. Avg, average temperature; Min, minimum temperature; Max, maximal temperature.

Table	*N*	Median Barefoot/Shod	Mean Barefoot/Shod	Standard Deviation Barefoot/Shod	Minimum Barefoot/Shod	Maximum Barefoot/Shod
Avg, dorsal	8	22.4/15.2	19.56/13.68	6.81/7.8	8.1/2.75	25.2/23.0
Min, dorsal	8	13.7/7.9	11.61/9.37	4.73/10.52	3.5/5.75	15.15/13.0
Max, dorsal	8	27.75/24.25	24.7/23.02	7.13/6.21	13.3/13.15	30.45/29.8
Avg, palmar	8	22.25/19.22	18.57/17.71	9.59/5.21	3.63/11.8	27.45/23.57
Min, palmar	8	14.07/7.8	15.12/7.9	8.26/2.86	4.47/5.3	30.0/12.6
Max, palmar	8	27.26/25.72	25.92/24.12	6.41/5.92	14.12/15.02	31.82/29.5

**Table 2:** Thermographic results received from group 2 before and after shoeing with wedge pads. Avg, average temperature; Min, minimum temperature; Max, maximum temperature.

Table	*N*	Median Barefoot/Shod	Mean Barefoot/Shod	Standard Deviation Barefoot/Shod	Minimum Barefoot/Shod	Maximum Barefoot/Shod
Avg, dorsal	8	22.95/20.4	20.94/19.27	5.17/2.89	8.7/12.3	25.2/21.55
Min, dorsal	8	13/9.12	12.09/9.22	2.92/1.96	4.5/5.05	14.4/12.55
Max, dorsal	8	29.02/27.42	26.3/26.44	6.53/3.47	11.3/17.1	31/28.95
Avg, palmar	8	25.58/22.23	23.79/21.53	5.47/2.82	10.55/14.6	28.07/23.9
Min, palmar	8	12.81/7.72	12.69/7.25	2.65/1.86	6.5/3.87	16.5/9.97
Max, palmar	8	30.16/28.31	28.28/28.11	5.45/2.72	14.12/21.52	31.85/30.55

## Data Availability

The datasets used and/or analyzed during the current study are available from the corresponding author upon reasonable request.
